# The role of serotonin hormone on weight loss maintenance after sleeve gastrectomy: a systematic review

**DOI:** 10.25122/jml-2023-0356

**Published:** 2024-02

**Authors:** Yaser Al Naam

**Affiliations:** 1 Prince Sultan Military College of Health Sciences, Dammam, Saudi Arabia

**Keywords:** serotonin, weight loss maintenance, laparoscopic sleeve gastrectomy, dietary factors, behavioral interventions, systematic review

## Abstract

Surgical interventions, such as laparoscopic sleeve gastrectomy (LSG), are frequently associated with significant weight loss. However, the initiation and maintenance of this weight reduction are multifaceted processes influenced by genetic, psychological, behavioral, dietary, and metabolic factors. This review examined the role of metabolic hormones, specifically serotonin, in sustaining weight loss post-LSG. A systematic evaluation of six research articles obtained from Scopus, PubMed, and Cochrane was conducted, focusing on the role of serotonin in weight loss maintenance. We included randomized controlled trials involving adults over 18 years. Studies lacking an intensive weight regulation approach were excluded. Information was systematically extracted and analyzed from the selected studies, with data on intervention and control groups summarized in tables to compare outcomes one year post-LSG. The findings revealed a complex interplay between serotonin and its role in weight maintenance after sleeve gastrectomy. While some studies demonstrated successful weight loss maintenance with serotonin intervention, the systematic review found no association between serotonin and weight loss maintenance. Factors beyond serotonin levels, including individual motivation, behavioral strategies, and physical activity, were identified as crucial contributors to sustained weight loss. While the results may not demonstrate a recognizable association between serotonin and weight loss maintenance, the significance of this review lies in its contribution to the existing body of knowledge. By synthesizing current evidence, the study adds a nuanced perspective to understanding factors influencing post-LSG outcomes.

## INTRODUCTION

The prevalence and steady rise of obesity over the past decades has become a global issue. Obesity leads to numerous severe health complications, including diabetes, certain cancers, and cardiovascular diseases, making it a critical public health issue [1]. Various weight loss interventions have been developed in response to this growing challenge. Among these, laparoscopic sleeve gastrectomy (LSG) has emerged as a key surgical intervention. This procedure, which involves the surgical removal of a portion of the stomach to decrease food intake and promote weight loss, has been demonstrated to be an effective tool in the fight against obesity [2]. However, while initial weight loss post-surgery can be significant, maintaining this weight reduction poses a substantial challenge for many patients [3]. Research by Mauro *et al*. indicates that many patients regain weight after some time [4]. Understanding the mechanisms behind long-term weight loss maintenance is essential to optimize the outcomes of sleeve gastrectomy.

Serotonin, a neurotransmitter with diverse functions in the body, plays a potential role in weight loss maintenance after SLG [5]. Beyond its well-known influence on mood and mental health, serotonin also impacts metabolism and food intake [6-8]. The multifaceted role of serotonin makes it an essential element in investigating weight loss maintenance after sleeve gastrectomy.

This systematic review aimed to investigate the role of serotonin in weight maintenance post-LSG by examining how variations in serotonin levels and its signaling pathways may influence weight regulation. This analysis is crucial for understanding whether different serotonin dynamics can effectively support weight maintenance, assess the impact of serotonin on weight regulation, and determine the potential of serotonin as a therapeutic target for improving long-term weight management outcomes in patients post-LSG. Furthermore, the review comprehensively explored the relationship between serotonin and weight loss maintenance by analyzing various studies. It also considered potential interventions to enhance weight maintenance strategies, such as dietary approaches targeting serotonin.

### Metabolic hormones and weight loss maintenance

Different organs and tissues within the body produce various metabolic molecules, which play essential roles in regulating metabolism, appetite, and energy [9,10]. After the performance of LSG, specific alterations in hormones are key to metabolic improvement and weight loss [11]. Hormones such as ghrelin, leptin, insulin, and peptide YY (PYY) are pivotal in sustaining weight loss post-LSG [12-14]. Notably, ghrelin, often referred to as the 'hunger hormone,' is primarily secreted by the stomach and stimulates appetite. LSG leads to a marked reduction in ghrelin levels, attributed to removing a portion of the stomach responsible for its production [15,16]. The decline leads to reduced hunger and improved hunger control among individuals, thus making the reduced calories in diet maintenance and subsequent weight loss easier.

Leptin, another essential hormone, is produced by adipose tissues and regulates appetite and energy use. In appetite regulation, leptin is a feedback mechanism from fatty tissues to the brain, where it acts on the hypothalamus [17]. The hypothalamus, which controls various physiological processes, including appetite, responds to leptin by binding to specific receptors that signal a feeling of satiety, thus suppressing appetite. Patients experience significant weight loss after LSG, significantly reducing leptin levels [18]. This decline is associated with decreased energy expenditure, a common outcome for individuals post-LSG. Concurrently, LSG has an immediate positive impact on glucose metabolism and insulin sensitivity, even before significant weight loss occurs [19]. This is partly due to the alteration of gut anatomy; LSG involves the reconfiguration of the small intestine and a reduction in stomach size, which changes the normal digestive process and promotes changes in insulin secretion and action [20]. Improved insulin function contributes to better glycaemic control and may help prevent weight regain by reducing the likelihood of post-meal blood sugar spikes and cravings [21]. Glucagon-like peptide-1 (GLP-1) and peptide YY also play a crucial role in weight loss maintenance [22]. The two hormones are released from the small intestine in response to food intake and play a significant role in appetite suppression and satiety. Following LSG, the reduced stomach volume and accelerated passage of food through the digestive tract result in an earlier release of PYY and GLP-1 [23]. This increases feelings of fullness and reduced appetite, promoting weight loss maintenance.

Individuals might experience significant challenges in weight loss maintenance post-LSG due to factors such as metabolic adaptation, dietary factors, and psychological elements. The body adapts to various metabolic changes and hormone regulations over time, potentially leading to weight gain [24]. Increases in ghrelin, for instance, can lead to increased appetite and, thus, weight gain. Furthermore, the extent to which patients adhere to dietary and lifestyle aspects varies since some may struggle to maintain regular exercise. In contrast, some may comfortably adhere to recommended lifestyles post-LSG [25]. Eating behaviors can further be influenced by psychological factors such as emotional eating, depression, and stress [26]. These elements pose significant challenges to sustaining weight loss in patients who have undergone LSG. Given the challenges associated with maintaining weight loss after LSG, various interventions and strategies have been developed to support patients' weight management efforts. Mendes *et al*. [27] highlight fundamental approaches, including psychological support, nutritional guidance, physical activity promotion, pharmacotherapy, and revisional surgery [28]. Counseling services are essential for helping patients manage emotional factors that can affect eating habits. This support aims to provide patients with healthier coping mechanisms for dealing with stress, depression, or emotional eating. In nutritional guidance, patients are exposed to meal planning and diet control guidelines, which help them develop sustainable eating habits [29]. For some patients, pharmacological interventions may be necessary. These involve prescribing medications that help regulate appetite and reduce cravings under careful medical supervision. Such initiatives help overcome the difficulties of weight loss maintenance.

### Serotonin and weight loss maintenance

Serotonin, commonly referred to as the “feel-good” neurotransmitter, plays a crucial role in the body that extends beyond mood regulation. Since the hormone influences the appetite of individuals, it is a key contributor to weight regulation [30]. When the brain signals for the release of serotonin, it triggers the activation of cells in the stomach responsible for producing this hormone. This activation occurs through the binding of serotonin to specific receptors known as 5-hydroxytryptamine receptor 2C (5-HT2C) located in the hypothalamus section of the brain [19]. Activated 5-HT2C ultimately reduces appetite and increases the feeling of satisfaction. As such, higher serotonin levels are associated with better appetite control, which is key in weight loss maintenance. Research indicates that laparoscopic sleeve gastrectomy can influence the production of serotonin, resulting in elevated levels of serotonin circulating in the body [31]. This increase is associated with changes in gut physiology, including the accelerated transit time of food through the digestive tract, prompting the release of serotonin hormone from enterochromaffin cells [32]. The heightened levels of serotonin post-LSG are believed to contribute to more effective appetite management and improved weight loss maintenance.

Beyond its role in appetite control, serotonin is crucial in regulating emotions and mental well-being. A decrease in serotonin levels is consistently linked to increased anxiety and depression, potentially leading to emotional eating and subsequent weight gain [33]. The ability of LSG to modulate serotonin sensitivity positively impacts patients' quality of life, reducing mood-related issues and, consequently, diminishing the likelihood of emotional eating. It is noteworthy that emotional eating can have dual effects on weight, as individuals experiencing depression may sometimes eat less, contributing to weight loss [34]. However, evidence suggests that emotional eating is more commonly associated with weight gain, as individuals may consume more in response to their emotions.

The emerging roles of serotonin in weight regulation and its impacts in post-LSG have created a growing interest in developing therapeutic strategies to enhance weight loss maintenance. Among the strategies developed include pharmacological interventions, dietary approaches, and lifestyle modifications [35]. Strategies that promote overall well-being, such as stress reduction, regular physical activity, and adequate sleep, can indirectly influence serotonin levels and mood. Incorporating these lifestyle changes post-LSG may contribute to better weight maintenance outcomes. Furthermore, dietary choices play a significant role in affecting serotonin production. Foods rich in tryptophan, the amino acid precursor to serotonin, can support serotonin synthesis. Thus, incorporating a balanced diet that includes such foods post-LSG could naturally enhance serotonin levels [36]. While research continues research on potential medications targeting serotonin receptors, some drugs, such as selective serotonin reuptake inhibitors (SSRIs), have shown effectiveness in managing appetite and mood, which are crucial for maintaining long-term weight loss [37]. Despite considering different post-surgical maintenance protocols, the effectiveness of such approaches has become challenging. The current research, therefore, aimed to investigate the role of serotonin in weight loss maintenance after LSG.

## MATERIAL AND METHODS

The research strategy for this systematic review followed a comprehensive approach, aiming to identify and cross-check relevant studies related to the role of serotonin in weight loss maintenance after LSG. Various databases, including Scopus, PubMed, and Cochrane, were extensively searched to ensure the relevance of the review. A combination of keywords was used, including 'serotonin', 'weight loss maintenance', 'laparoscopic sleeve gastrectomy', 'dietary factors', and 'behavioral interventions'. The selection process involved screening titles, reviewing abstracts, and evaluating full texts, with expert consultation for additional data clarification when necessary.

### Inclusion and exclusion criteria

**Figure 1:**
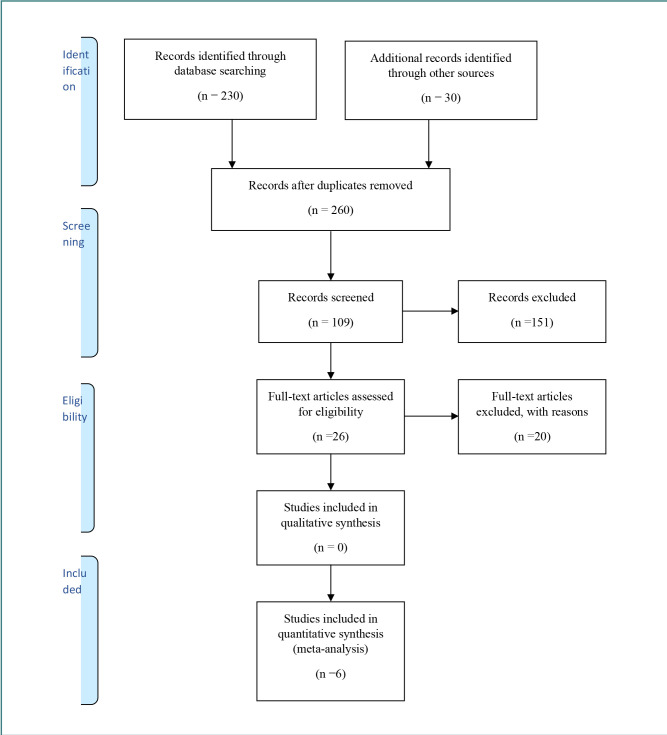
PRISMA flow diagram

This systematic review included randomized controlled trials (RCTs) to explore the role of serotonin in weight loss maintenance after LSG. To ensure relevance and reliability, the selection criteria included factors such as study design, participant demographics (age and gender), dietary treatments, behavioral approaches, control groups, and data suitability for analysis. The search process followed the PRISMA model proposed by Moher *et al*. (Figure 1) [34]. RCTs were prioritized due to their rigorous design, which minimizes bias and strengthens the reliability of the findings in this review. To ensure the generalizability of our findings to adult populations, we included participants aged 18 and above, as the impact of serotonin may vary across different age groups [35,38]. Participants with a history of dietary interventions before undergoing LSG were included to establish a uniform baseline for weight loss efforts, enhancing the comparability of study outcomes. Exclusion criteria were established for studies that did not implement an intensive weight regulation approach during the maintenance phase. Intensive weight regulation was considered crucial for maximizing weight preservation in previous steps. Studies without control groups were excluded, as comparisons between intervention and control groups are essential for effective outcome comparison. Furthermore, studies with insufficient data for analysis, such as missing weight values or changes, were also excluded. Only English-language, peer-reviewed articles published within the last 5 years were considered. Duplicate studies, those without interventions or lacking a surgical context, were also excluded.

### Data extraction and quality assessment

The next step involved data extraction and quality assurance after carefully selecting studies that met the inclusion criteria. Ensuring that data extraction adhered to quality principles was necessary to increase the reliability and validity of the results. Information was systematically collected from the six included studies, focusing on key characteristics of the participants. These characteristics included the total number of patients, gender, and body weight. To provide a clear understanding of the overall study population, a table was created summarizing the sample size (number of participants) from each study. Gender distribution was also included in the table to allow for analysis of potential gender-related differences in outcomes. Detailed analysis of body weight data was performed to compare pre-and post-intervention values for both groups. Results are presented in a table for clear visualization. To ensure the trustworthiness of the data, the Critical Appraisal Skills Programme (CASP) checklist was used to rigorously assess the quality of the included studies. CASP is a widely recognized tool that provides a structured framework for assessing the methodological robustness of various study designs, ensuring a comprehensive evaluation process [39]. The CASP checklist guided a systematic appraisal of each study, focusing on potential bias, confounding variables, and the overall validity of the research design. The assessment aimed to identify potential sources of bias that could impact the reliability of the findings. Additionally, the checklist facilitated a detailed examination of the clarity and transparency of the study methodologies, enhancing the researcher's ability to make informed judgments about the internal and external validity of the evidence.

## RESULTS

The comprehensive literature search yielded 260 articles relevant to the role of serotonin in weight loss maintenance after LSG (Figure 1). Initially, 230 articles were identified through systematic database searches, and an additional 30 records were found via other sources, such as conference proceedings. After deduplication, 260 unique articles remained. Screening of titles and abstracts narrowed this number to 109 articles, with 151 excluded for not meeting our predefined criteria. Subsequently, a thorough full-text assessment was conducted, resulting in the exclusion of 20 articles due to a lack of relevant outcomes or methodological coherence. Six studies were included for in-depth analysis.

### Characteristics of studies included in the systematic review

This systematic review included data from six studies on the role of serotonin in weight loss maintenance after LSG surgery (Table 1). A total of 2,312 patients were analyzed, with 598 female participants (representing approximately 26% of the sample). The participant demographics reflected a diverse age range, with a mean age ranging between 41 and 65 years old.

**Table 1:** Characteristics of studies included

Name of Author	Year	Participants	Intervention Group
Nikiforova *et al*. [40]	2019	500	Dietary changes in relation to weight loss monitored
Shenhar *et al*. [41]	2019	1527	Routine annual check-up
Xiong *et al*. [42]	2021	124	Monitoring of transcutaneous electrical acupoint stimulation
Williams *et al*. [43]	2019	108	Nutritional consultations
Sniehotta *et al*. [44]	2019	511	Portal for weight entry
Nakata *et al*. [45]	2019	154	Monitoring weight on a daily basis

The six studies included in this review implemented diverse protocols to examine the role of serotonin in weight loss maintenance after LSG surgery (Table 2). Nikiforova *et al*. [40] conducted a three-year follow-up monitoring weight loss and eating habits, while Shenhar *et al*. [41] focused on weight monitoring during routine check-ups with additional health assessments. Xiong *et al*. [42] investigated short-term weight loss within 48 hours post-surgery using a specific stimulation technique. Intervention approaches varied across the remaining studies. Williams *et al*. [43] compared an intervention group receiving consultations with a nutritionist and sports physiologist to a control group receiving only paper materials [43]. Sniehotta *et al*. [44] employed a unique protocol with daily weight entry, monitored physical activity, and food diaries. The intervention group in this study received personalized text message feedback, while the control group received general suggestions less frequently. Similarly, Nakata *et al*. [45] provided web support for weight and physical activity monitoring to the intervention group post-weight loss, while the control group did not receive additional support. The weight values at the beginning of the study (T0) and after treatment (T12) were recorded for both the intervention and control groups across the six studies (Table 2).

**Table 2:** Maintenance interventions included in the systematic review

Author	Patients in the study			Age (Mean ± SD)		Intervention (Mean ± SD)		Control (Mean ± SD)	
	Total	Treated	Control	Treated	Control	T0	T12	T0	T12
Nikiforova *et al*. [40]	300	300	300	41.65 ± 11.05	41.65 ± 11.05	117.83 ± 17.63	117.13 ± 14.4	117.83 ± 17.63	113.3 ± 13.4
Shenhar *et al*. [41]	1527	1450	77	52.13 ± 11.75	51.18 ± 14.22	91.57± 13.8	84.57 ± 13.2	90.2 ± 12.9	82.2 ± 11.7
Xiong *et al*. [42]	62	31	31	61.5 ± 8.3	62.0 ± 8.3	100.2 ± 19.5	93.2 ± 13.9	100.2 ± 21.7	95.2 ± 14.8
Williams *et al*. [43]	54	28	26	47.3 ± 1.8	47.3 ± 1.8	68.7 ± 8.9	65.6 ± 8.5	68.6 ± 6.7	67.4 ± 6.7
Sniehotta *et al*. [44]	288	144	144	42.0 ± 11.6	41.6 ± 11.4	85.1 ± 17.5	86.8 ± 18.2	85.2 ± 15.7	87 ± 16.7
Nakata *et al*. [45]	95	47	48	54.7 ± 6.6	57 ± 5.7	74.7 ± 10.6	69 ± 10.9	74.2 ± 8.1	67.7 ± 8.7

SD represents the standard deviation of the age and weight values of participants

The study by William *et al*. [43] and Shenhar *et al*. [41] implemented intensive treatment approaches during the maintenance phase, significantly reducing weight. As seen in Table 2, patients with intensive treatment had a substantial reduction in weight loss. For example, Williams *et al*. [43] reported an over 3 kg weight loss in the intervention group compared to the control group, which had 1.2 kg. Similarly, Shenhar *et al*. [41] observed a nearly 7 kg weight loss in the intervention group compared to the control group, which was 5 kg. These intensive protocols often involved regular group sessions and consultations with healthcare professionals. However, these studies did not directly assess the role of serotonin post-LSG. Some studies focused on other hormones, such as acetylcholinesterase (AChE). Consequently, there was no clear conclusion on the role of serotonin, thus creating a gap for further studies on its role in weight loss maintenance after LSG.

Compared to the intensive approaches employed by Williams *et al*. [43] and Shenhar *et al*. [41], the protocols implemented by Nakata *et al*. [45] and Xiong *et al*. [42] were more moderate. Nakata *et al*. [45] and Xiong *et al*. [42] had significant weight reductions in both intervention and control groups, even though Xiong *et al*. [42] had an effect that was relatively higher in the control group (8 kg) compared to the intervention group (7 kg). Similarly, Nakata *et al*. [45] reported a 6 kg reduction in the control group compared to 5 kg in the intervention group. Their treatment included daily self-monitoring of weight by participants. Although these studies included prophylactic antiemetics, which might influence serotonin levels, none directly investigated the role of serotonin itself.

Sniehotta *et al*. [44] and Nikiforova *et al*. [40] used maintenance protocols in their studies, highlighting the challenges of sustaining weight loss. Generally, patients who followed more intensive approaches experienced reduced weight regain. Sniehotta *et al*. [44] reported a weight gain of 1.8 kg in the control group compared to 1.7 kg in the intervention group, with both groups performing daily self-monitoring. Similarly, Nikiforova *et al*. [40] observed a weight gain of 4.5 kg in the control group, compared to only 0.7 kg in the intervention group. While these studies effectively analyzed weight loss maintenance after LSG, none provided a clear role for the serotonin hormone. Table 3 presents a detailed data quality assessment based on the CASP criteria across selected studies.

**Table 3:** Data quality assessment

CAS Question/Article	Nikiforova *et al*. [40]	Shenhar *et al*. [41]	Xiong *et al*. [42]	Williams *et al*. [43]	Sniehotta *et al*. [44]	Nakata *et al*. [45]
Did the review address a focused question?	Yes	Yes	Yes	Yes	Yes	Yes
Did the authors look for the right type of papers?	Yes	Yes	Yes	Yes	Yes	Yes
Were all the important, relevant studies included?	Yes	Yes	Yes	Yes	Yes	Yes
Did the review’s authors do enough to assess the quality of the included studies?	Yes	Yes	Yes	Yes	Yes	Yes
If the review results have been combined, was it reasonable to do so?	Can’t tell	Can’t tell	Can’t tell	Can’t tell	Can’t tell	Can’t tell
What are the overall results of the review?	By the objectives	By the objectives	By the objectives	By the objectives	By the objectives	By the objectives
How precise are the results?	Precise	Highly Precise	Moderately Precise	Precise	Highly Precise	Highly Precise
Can the results be applied to the local population	Yes	Yes	Yes	Yes	Yes	Yes
Were all important outcomes considered?	Yes	Yes	Yes	Yes	Yes	Yes
Are the benefits worth the harms and costs	Yes	Yes	Yes	Yes	Yes	Yes

## DISCUSSION

This systematic review intended to assess the role of serotonin hormone in weight variations after LSG and the effectiveness of various post-surgical maintenance protocols. The analysis included six studies with a minimum follow-up duration of 12 months. The findings from this review present a nuanced perspective. While some studies demonstrated improved outcomes in intervention groups compared to control groups, there was no relationship between serotonin hormone and weight loss maintenance. This suggests that even with intensive intervention approaches during maintenance, maintaining previously achieved weight loss may remain challenging [46].

These results emphasize the complexity of long-term weight management and suggest that factors beyond intensive behavioral treatment play a crucial role. Considerations include individual motivation, behavioral approaches, gender-related differences, and the nature of physical activity during weight reduction and maintenance phases. Further research is warranted to explore the multifaceted factors of serotonin hormone influencing weight variations following LSG. A deeper understanding of the interplay between the serotonin hormone, behavioral interventions, and patient-specific factors will contribute to more effective strategies for sustaining weight loss outcomes in this population.

The systematic review found that alterations in plasma tryptophan levels can influence brain tryptophan levels and, subsequently, serotonin concentrations. For instance, Shenhar *et al*. [41] revealed a significant decrease in serum and brain tryptophan levels within hours of taking a tryptophan-deficient diet. Conversely, insulin injection increased serotonin levels, with dietary components like fatty acids, carbohydrates, and proteins contributing to elevated insulin secretion and increased plasma tryptophan levels. This rise in plasma tryptophan leads to increased brain tryptophan, increasing brain serotonin concentration [47]. Diet plays a significant role in regulating serotonin levels. High-fat and high-sugar diets can decrease serotonin transporter (SERT) binding, potentially lowering serotonin levels [48]. For instance, the ketogenic diet (KD), characterized by low carbohydrates and high fats, can influence norepinephrine, dopamine, and serotonin concentrations [49]. These dietary patterns, often associated with frequent snacking, may disrupt the serotonergic system and increase the risk of obesity. Understanding the relationship between dietary components and serotonin provides valuable insights for clinical applications. Food items known to raise serotonin levels can be incorporated into patient diets to modulate brain outputs associated with serotonergic neurons [50]. Additionally, knowledge of this relationship can enhance drug efficacy in serotonin-receptor interactions. Conversely, tryptophan-deficient diets can potentially boost the effectiveness of 5-HT-blocking drugs.

The results presented in this review highlight the challenges associated with maintaining weight loss following LSG. The role of serotonin in this context is multifaceted. The influence of serotonin on appetite regulation and mood suggests that it may play a role in the long-term success of weight loss interventions [51]. However, the efficacy of intensive behavioral approaches in maintaining weight loss has yielded mixed results. Factors such as individual motivation, behavioral strategies, gender-related differences, and the type of physical activity during the weight reduction and maintenance phases likely contribute to the varying outcomes observed in weight maintenance protocols [52].

### Limitations

Despite the rigorous methodology employed in this systematic review, it is crucial to acknowledge specific limitations that influenced the interpretation and generalizability of the findings. The quality and availability of the selected sources, especially the reliance on published literature and exclusion of unpublished literature, introduced publication bias since studies with significant findings were likely to have been published. Excluding non-English studies introduces language bias and potentially overlooks relevant research. The heterogeneity in study methods, interventions, outcome measures, and participant characteristics limited the ability to draw unified conclusions due to challenges in synthesizing results across studies. Moreover, including studies with different follow-up durations introduced variability in assessing long-term weight loss maintenance outcomes after LSG.

## CONCLUSION

This systematic review analyzed the role of serotonin in weight loss maintenance after LSG and its relationship with dietary factors. The review identified key aspects of weight maintenance and the complex relationships between the serotonin hormone and long-term weight maintenance. Studies included in the review revealed diverse outcomes related to the effectiveness of different maintenance protocols following LSG. While some studies demonstrated substantial weight loss maintenance with intensive interventions, others faced challenges sustaining the achieved weight loss. The systematic review did not yield statistically significant results, underscoring the complexity of this field and the need for further research. The studies provided no apparent role of serotonin hormone in weight loss maintenance. Being an essential contributor to appetite regulation, it is apparent that serotonin plays a role in the long-term management of body weight. The varying outcomes observed in weight maintenance protocols showed that some factors, such as physical activity, may be responsible for sustaining weight loss.

The review analyzed vast amounts of information from the resources despite certain limitations, including protocol variations, human body response complexities, and duration of follow-ups. Personalized approaches to weight maintenance are needed due to the human body's complexities. It is recommended that future research consider specific aspects in which weight loss maintenance is affected by serotonin. Moreover, individualized factors in successful long-term weight loss maintenance should be considered.
